# Immunoglobulin class‐switch recombination: Mechanism, regulation, and related diseases

**DOI:** 10.1002/mco2.662

**Published:** 2024-08-13

**Authors:** Jia‐Chen Liu, Ke Zhang, Xu Zhang, Fei Guan, Hu Zeng, Masato Kubo, Pamela Lee, Fabio Candotti, Louisa Katherine James, Niels Olsen Saraiva Camara, Kamel Benlagha, Jia‐Hui Lei, Huamei Forsman, Lu Yang, Wei Xiao, Zheng Liu, Chao‐Hong Liu

**Affiliations:** ^1^ Institute of Hematology, Union Hospital, Tongji Medical College, Huazhong University of Science and Technology Wuhan China; ^2^ Department of Pathogen Biology School of Basic Medicine, Tongji Medical College and State Key Laboratory for Diagnosis and Treatment of Severe Zoonotic Infectious Diseases, Huazhong University of Science and Technology Wuhan Hubei China; ^3^ Department of Respiratory The First Affiliated Hospital of Yangtze University Jingzhou China; ^4^ Department of Immunology Mayo Clinic College of Medicine and Science Rochester USA; ^5^ Laboratory for Cytokine Regulation, Center for Integrative Medical Science (IMS), RIKEN Yokohama Institute Yokohama Japan; ^6^ Department of Paediatrics and Adolescent Medicine LKS Faculty of Medicine The University of Hong Kong Hong Kong China; ^7^ Division of Immunology and Allergy Lausanne University Hospital and University of Lausanne Lausanne Switzerland; ^8^ Centre for Immunobiology Blizard Institute, Queen Mary University of London London UK; ^9^ Department of Immunology Institute of Biomedical Sciences, University of São Paulo São Paulo Brazil; ^10^ Institut de Recherche Saint‐Louis Université de Paris Paris France; ^11^ Department of Rheumatology and Inflammation Research Institute of Medicine, Sahlgrenska Academy, University of Gothenburg Gothenburg Sweden; ^12^ Department of Otolaryngology‐Head and Neck Surgery Tongji Hospital, Tongji Medical College, Huazhong University of Science and Technology Wuhan China

**Keywords:** antibody diversification, B cell development, genetic defects, immunodeficiency, isotype switching

## Abstract

Maturation of the secondary antibody repertoire requires class‐switch recombination (CSR), which switches IgM to other immunoglobulins (Igs), and somatic hypermutation, which promotes the production of high‐affinity antibodies. Following immune response or infection within the body, activation of T cell‐dependent and T cell‐independent antigens triggers the activation of activation‐induced cytidine deaminase, initiating the CSR process. CSR has the capacity to modify the functional properties of antibodies, thereby contributing to the adaptive immune response in the organism. Ig CSR defects, characterized by an abnormal relative frequency of Ig isotypes, represent a rare form of primary immunodeficiency. Elucidating the molecular basis of Ig diversification is essential for a better understanding of diseases related to Ig CSR defects and could provide clues for clinical diagnosis and therapeutic approaches. Here, we review the most recent insights on the diversification of five Ig isotypes and choose several classic diseases, including hyper‐IgM syndrome, Waldenström macroglobulinemia, hyper‐IgD syndrome, selective IgA deficiency, hyper‐IgE syndrome, multiple myeloma, and Burkitt lymphoma, to illustrate the mechanism of Ig CSR deficiency. The investigation into the underlying mechanism of Ig CSR holds significant potential for the advancement of increasingly precise diagnostic and therapeutic approaches.

## Graphical abstract

CSR switches IgM to other immunoglobulins. Following immune response or infection within the body, activation of T cell‐dependent and T cell‐independent antigens triggers the activation of AID, initiating the CSR process. CSR has the capacity to modify the functional properties of antibodies, thereby contributing to the adaptive immune response in the organism.

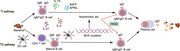

## 1 INTRODUCTION

In the natural environment, the human body encounters a complex array of foreign antigens. By developing a highly diverse range of antigen receptors, the acquired immune system functions to identify and eliminate potential pathogens. The generation of antigen‐specific antibodies is a hallmark of humoral immunity, which occurs as a result of programmed DNA breakage, rearrangement, or mutations. B‐cell‐specific activation‐induced cytidine deaminase (AID) initiates antibody CSR. The mechanism and biological function of AID have been fully elaborated.^1^, ^2^ However, the molecular pathways that regulate the production and diversification of different antibody isotypes remain of interest. Therefore, it is necessary to explore the molecular pathogenesis of typical diseases associated with antibody diversification.

CSR refers to the reaction that occurs after immunization or infection in vivo, which causes antigen‐activated IgM‐expressing B cells to undergo a change in the constant domain of the Ig μ heavy chain (HC).^3^ This alteration can be induced by T cell‐dependent activation or T cell‐independent antigen activation, leading to the generation of new isotypes and playing a crucial role in adaptive immune responses by altering the effector functions of antibodies.^3^ Ig CSR is a process of gene rearrangement. By altering the constant region of the Ig HC, B lymphocytes are able to undergo class switching of Ig from IgM to IgG, IgA, or IgE.^4^ At the level of DNA, CSR operates through the generation and joining of DNA double‐strand breaks (DSBs) at the switch regions located upstream of each HC constant region.^4^

CSR is an important part of the immune diversification function of an organization. The BCR isoforms possess the ability to switch from IgM to other classes, consequently altering the effector function of specific BCRs and initiating diverse downstream immune responses.^5^ Furthermore, extensive research has demonstrated that in mice and humans, CSR of IgD is a rare event that occurs only in a few B cell subsets within distinct lymphoid tissues, including mesenteric lymph nodes, the peritoneal cavity, and mucosa‐associated tissues.^6^, ^7^ Recent studies have indicated that the CSR of IgD is triggered by microorganisms, suggesting the involvement of IgD in the regulation of microbial homeostasis.^8^

This review provides a comprehensive review of the molecular pathogenesis of typical diseases associated with antibody diversification imbalance. It discusses insights into the diversification of five Ig isotypes and provides a comprehensive overview of the significance of CSR in various diseases. The review begins by discussing the background of CSR research, including its definition, basic knowledge, and its important role and significant research implications for the body. Subsequently, the review introduces the process of B cell development and differentiation, the generation of specific antibodies, and the classical and regulatory mechanisms of CSR, which lay the theoretical foundation for the subsequent introduction of relevant diseases. In the section related to diseases associated with CSR, the review sequentially introduces diseases related to the imbalance of IgM, IgD, IgA, IgE, and IgG, as well as hematological diseases related to off‐target Ig CSR. Furthermore, the review elucidates the impact of Ig CSR on diseases and future directions. Finally, the review summarizes the research achievements and future prospects of CSR.

## 2 MECHANISM OF IG CSR

Antibodies are classified into various types, with CSR playing a crucial role in antibody diversification. This section will provide an overview of the structure and function of antibodies, the mechanism of CSR, and its role in combating bacterial and viral infections.

### 2.1 Structure and function of the Ig

Antibodies are a crucial component of the Ig superfamily and play a significant role in the humoral immune response within the body.^9^ During the initial stage of the innate immune response, antigen‐presenting cells recognize pathogens and present processed antigens to B cells, leading to somatic hypermutation (SHM) and clonal selection of B cells, ultimately resulting in their differentiation into plasma cells and the production of antibodies specific to the antigen.^10^, ^11^, ^12^, ^13^, ^14^, ^15^ The composition of Ig consists of two identical HCs and two identical light chains (LCs) connected by disulfide bonds.^16^ Each Ig molecule contains one receptor binding fragment (Fc, crystallizable fragment) and two antigen binding fragments (Fab).^17^ According to the different regions of the Ig H chain, Ig is classified into five isotypes, namely IgM, IgG, IgA, IgE, and IgD.^18^ IgM typically exists in the form of pentamers or hexamers composed of monomers with a size of approximately 190 kDa. Each monomer consists of HC and LCs, with the HC containing five structural domains, namely Vμ, Cμ1, Cμ2, Cμ3, and Cμ4, and the LC containing two structural domains, namely Vκ‐cκ or Vλ‐Cλ.^19^, ^20^ IgM is the first isotype produced before class switching and is capable of effectively recognizing and clearing pathogens in the early stages of immune defense.^21^ The natural IgM antibody, due to its flexible antigen‐binding region and pentameric structure, is capable of nonspecifically binding to a variety of microorganisms, allowing it to capture pathogens in lymphoid organs and exert immune regulatory functions.^22^, ^23^ IgG is composed of two identical heavy chains and two identical light chains connected by interchain disulfide bonds. IgG is the predominant serum isotype and the only one capable of crossing the placental barrier to provide immunity to the fetus.^24^ The structure of IgA differs from that of IgG in that it lacks interchain disulfide bridges within the hinge region, which may allow for independent bending of the IgA hinge sequences and potentially increase sensitivity to protein hydrolysis.^25^ IgA is present on the mucosal surfaces of the gastrointestinal, respiratory, and genitourinary tracts, playing a crucial role in mucosal immunity.^26^, ^27^ The specific function of IgD remains unclear. Some studies suggest that IgD produced by IgM^−^IgD^+^ plasma cells exhibits high specificity for respiratory parasites and pathogens.^6^ Other research indicates that IgD may protect B cells from tolerance.^28^ Further investigation is needed to fully comprehend its role.

IgE is a potent activator of the immune system, enhancing effector functions and antigen presentation. In addition to its involvement in allergic diseases and allergic reactions, IgE also plays a role in immune protection against parasites. It is also involved in triggering inflammatory cascade reactions, leading to vasodilation and enhanced local protective responses.^29^, ^30^, ^31^

**FIGURE 1:**
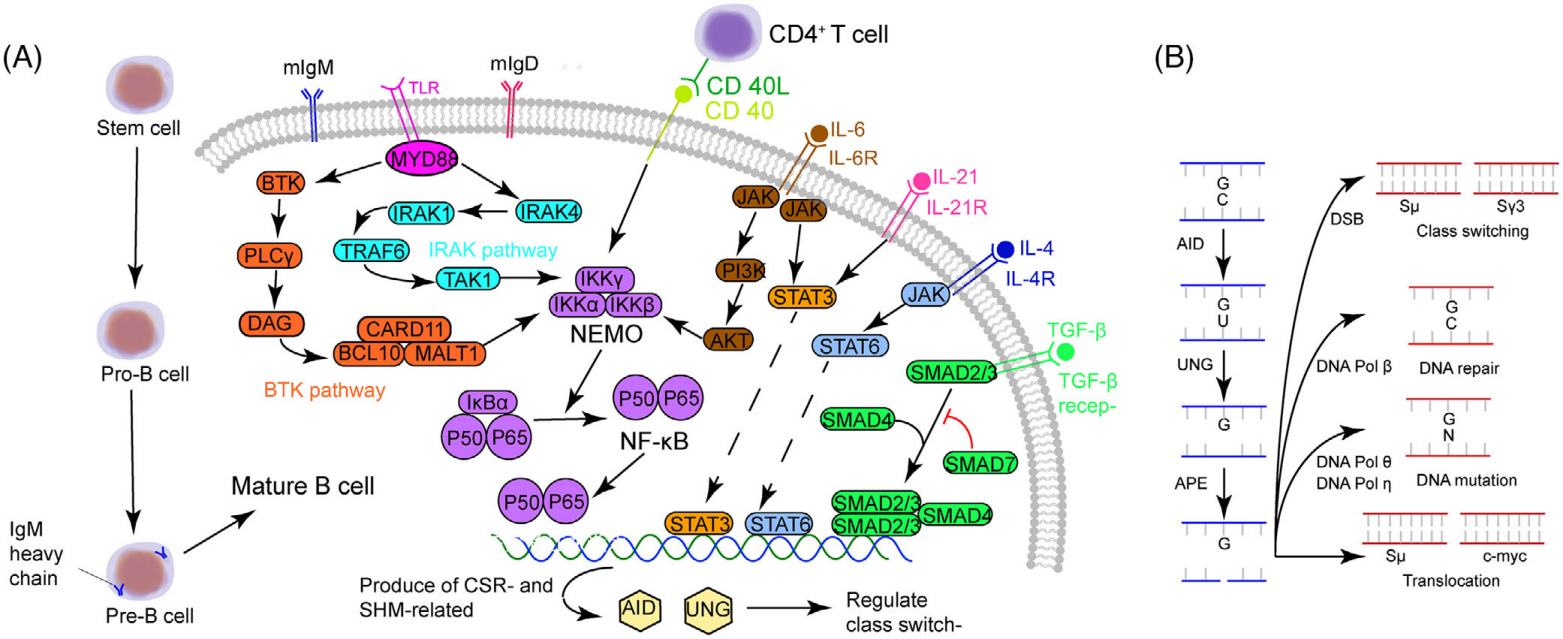
Pathogenesis of CSR deficiencies. CD40 interacts with CD40L and then activates downstream NEMO. NEMO removes the inhibitory subunits IκBα and releases reactive NF‐κB. NF‐κB is localized to the cell nucleus and regulates Ig production and CSR. The binding of TLR and MYD88 initiates two independent pathways, IRAK and BTK pathways. the former recruits IRAK1–4 and the later recruits PLCγ, DAG, and CARD11–BCL10–MALT1 complex, both pathways ultimately activate NEMO. Cytokines also affect Ig CSR. Upon binding with IL‐6R, IL‐6 initiates the JAK–PI3K–AKT signaling pathway, which then activates NEMO and contributes to Ig CSR. IL‐6 and IL‐21 both can activate STAT3 and enter the cell nucleus to regulate gene expression. Besides, IL‐4 has a JAK–STAT6 signaling pathway and exhibits a similar function as IL‐6 and IL‐21. TGF‐β activates SAMD2/3 and recruits SAMD4 to exert its function (Figure 2B). Schematic diagram of DNA breakage in the S region and the following genetic events including CSR, repairment, mutation, and translocation. AID initiates the deamination of cytosine to uracil in the S and IgV region, then UNG removes mismatched uracil and APE creates single‐stranded breaks (SSBs). When these SSBs escape from DNA repair, CSR, point mutation, and translocation happen. IKK, IκB kinase; TLR, toll‐like receptor; MYD, myeloid differentiation primary response protein; IRAK, interlukin‐1 receptor‐associated kinase; TRAF, TNF receptor‐associated factor; TAK, TGF‐activated kinase; BTK, Bruton's tyrosine kinase; PLC, phospholipase C; DAG, diacylglycerol; CARD, caspase recruitment domain‐ containing protein; MALT1, mucosa‐associated lymphoid tissue lymphoma translocation gene 1; JAK, Janus kinase; PI3K, phosphatidylinositol 3‐kinase; STAT, signal transducer and activator of transcription protein; SMAD, small mother against decapentaplegic protein; AID, activation‐induced deaminase; UNG, uracil DNA glycosylase; APE, apurinic/apyrimidinic endonuclease.

A single B cell produces an antibody with a single specificity, which is determined by the nature of VJ (L‐chain) and VDJ (H‐chain) rearrangements.^32^ In the early phases of B‐cell development, these rearrangements happen in a stochastic genetically programmed recombination. Hematopoietic stem cells differentiate into B lymphocytes early in the fetus’ development. The bone marrow is the major location of this early phase of B‐cell differentiation later in fetal development and for the remainder of one's life. As a result, the primary lymphoid organ for B‐cell development in humans is the bone marrow, which is also the place where the antigen‐specific receptor is first produced. Pro‐B cells are the first identifiable cells in the B cell lineage, displaying the first Ig gene rearrangement: a D_H_ gene segment rearranges to a J_H_ gene segment at the IgH‐chain locus. The IgH‐chain locus undergoes a second rearrangement in the pre‐B cell stage, when a V_H_ gene segment is recombined with the D_H_J_H_ segments, generating a unique VDJ unit.^33^ As a result, pre‐B cells synthesize a μ chain because genetically, the rearranged VDJ is located immediately upstream of C_μ_. L‐chains couple with μ chains to generate monomeric IgM, which is expressed at the cell surface in combination with Igα/Igβ to create the BCR in the next stage of B‐cell development, the immature B cells. The IgM produced on the surface of immature B cells can bind and respond to a single antigenic epitope, indicating that immature B cells can respond to antigens. Due to the essentially stochastic nature of IgM production, some immature B cells recognize self‐antigens, while others express specificity for foreign antigens. B cells will then undergo negative selection which reduces the number of self‐reactive B lymphocytes. Transitional B (TrB) cells are immature B cells that have survived negative selection in the bone marrow and have started to produce IgD alongside IgM on the cell surface through alternative splicing. After about 24 h in the bone marrow, those IgM^+^IgD^+^ mature B cells appear in the periphery^34^ (Figure 1A).

After migrating from bone marrow to peripheral blood, and then to the peripheral lymphoid tissue, mature B cells can encounter and be stimulated by antigens.^35^ The precursor of mature B cells is considered to be TrB cells, which are immature B cells originating from hematopoietic stem cells in the bone marrow.^36^, ^37^ Immature B cells migrate from the bone marrow to the periphery and undergo differentiation.^38^ During the differentiation process from TrB cells to mature naïve B cells, there is a selection for self‐reactive antibodies, leading to the gradual acquisition of immune competence in the human body.^39^, ^40^ Somatic mutations in the V genes of the H‐ and L‐chains during the lifespan of a B cell create high‐affinity B cell receptors (BCRs) to combat a variety of antigens. By competitively binding to antigens, those B cells with the highest affinity survive. In addition, with the help of cytokines and other immune cells, antibody CSR converts IgM to other Igs, including IgA, IgD, IgE, and IgG. Mature B cells reach a crossroad in the germinal center (GC) from which they can either migrate into the blood as memory cells or undergo differentiation to plasma cells.

The diversification of antibodies is a crucial process in the body's immune system, in which CSR plays an indispensable role. It induces antibody class switching, enabling the body to acquire immunity. Its specific mechanism is worthy of our research.

### 2.2 Classic mechanisms of CSR

In order for antibody‐mediated immune responses to be successful, the process of CSR is necessary.^41^ CSR involves the rearrangement of genes responsible for Ig production. B cells undergo changes in the constant region of the Ig HC, resulting in the production and connection of DSBs in the intron switching region located upstream of each HC constant region. This process allows for the switch from IgM to IgG, IgA, or IgE. CSR is achieved by splicing and rejoining the DNA. Recombination occurs between two S regions and a lariat‐shaped intermediate is generated and removed. The S region, which is situated upstream of all CH genes except Cδ, spans a length of between 1 and 10 kb depending on the isotype.^42^ The introduction of DSBs into the donor Sμ (Sμ) region results in recombination between this region and the downstream/receiver S region located 65–160 kb downstream.^43^ Then, the rearranged VDJ fragment is placed just upstream of a new constant region, and Igs that have the same variable region but different constant regions are produced.^33^ The AID initiates the process of SHM and CSR. AID plays a crucial role in the conversion of cytidine to uracil in the S region and IgV region, which is essential for both SHM and CSR processes in somatic cells.^44^

**FIGURE 2:**
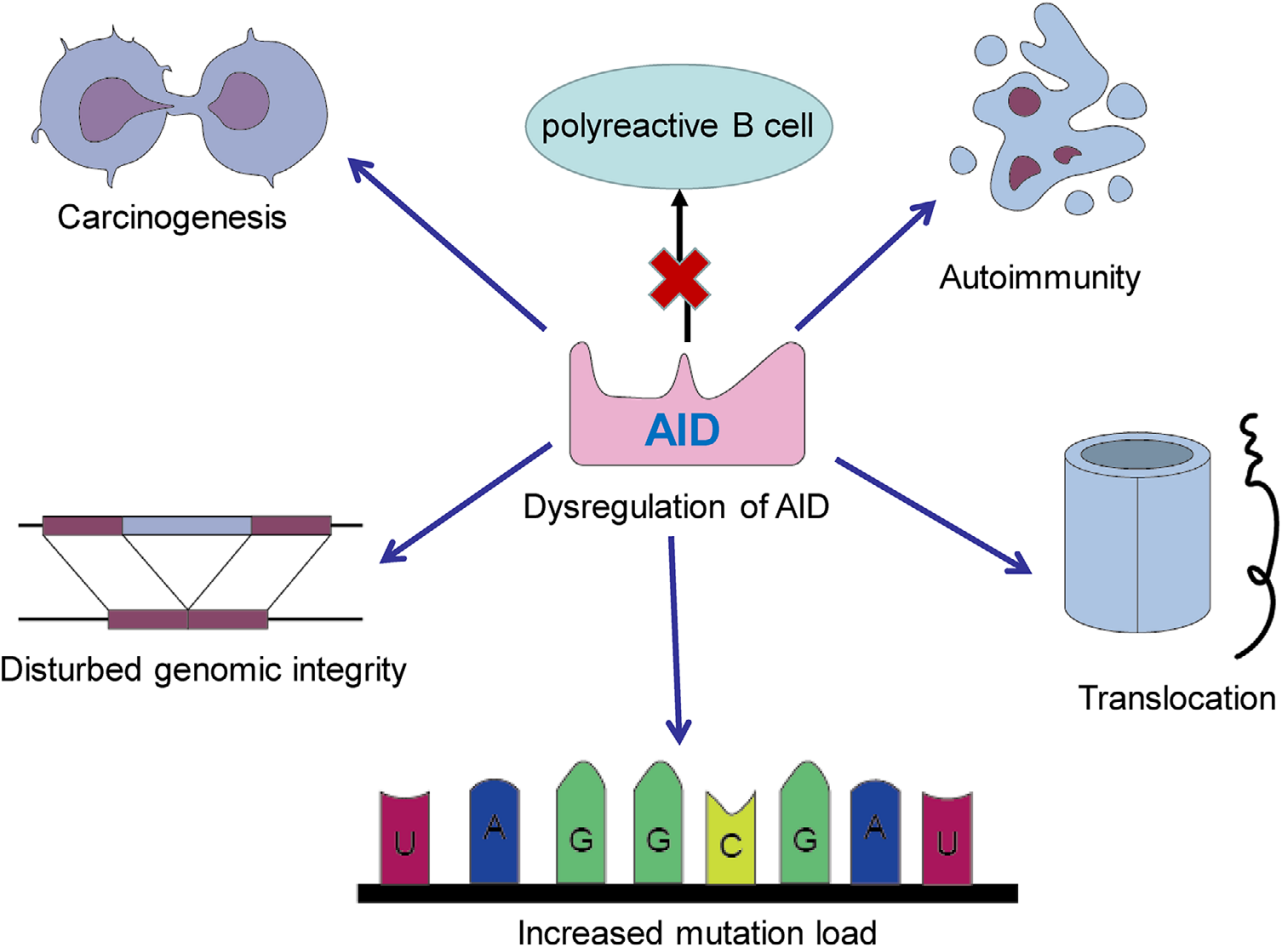
The dysregulation of AID can result in an increased mutational burden, translocation events, genomic instability, and the development of lymphoma. It also exhibits a certain inhibitory effect on polyreactive B cells.

**FIGURE 3:**
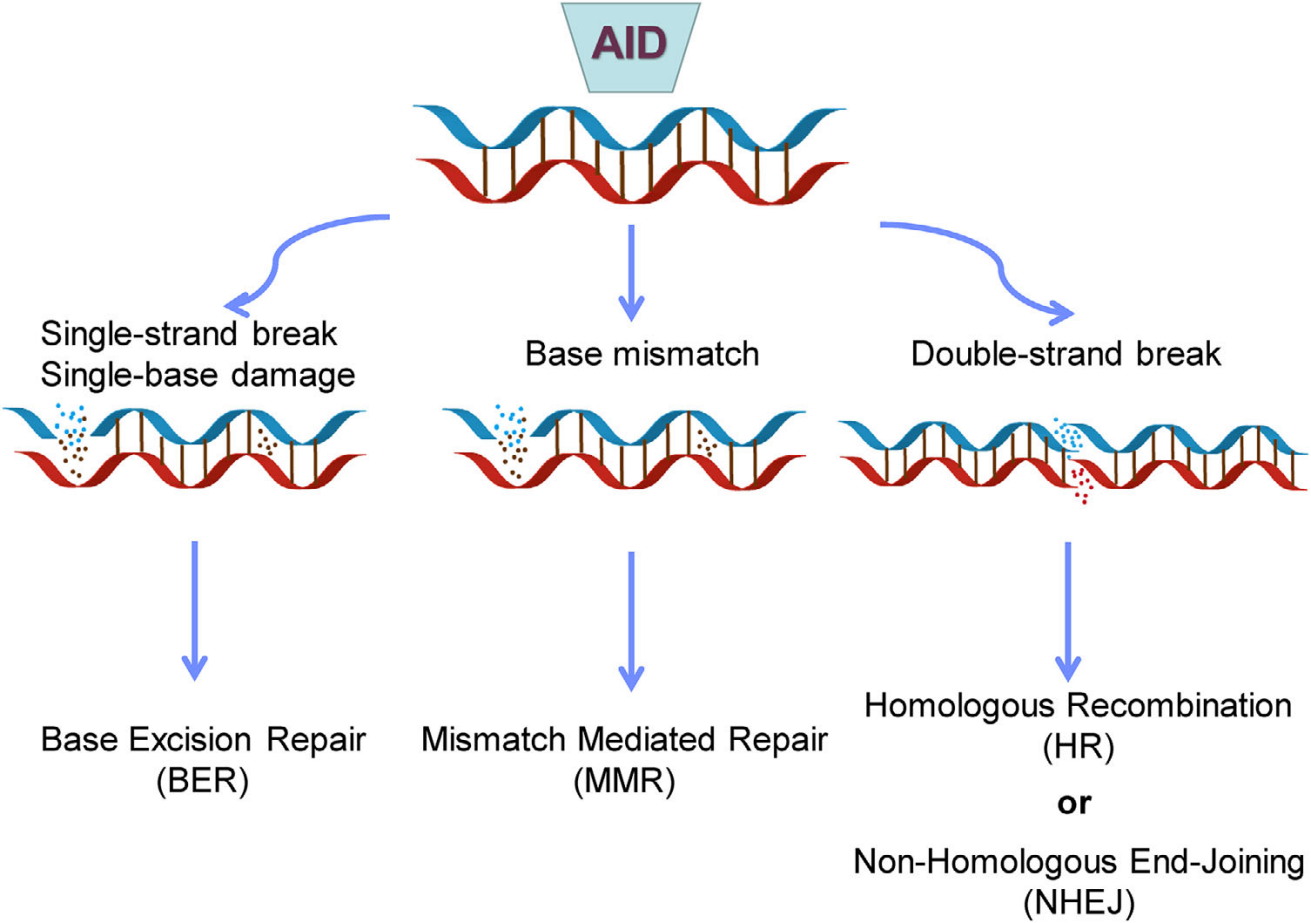
AID enzyme generates uracil, which then undergoes the base excision repair (BER) pathway, or is recognized as U:G mismatch and subsequently enters the mismatch repair (MMR) pathway. During the repair of uracil residues, single‐strand DNA breaks (SSB) are generated, which then convert to double‐strand DNA breaks (DSB), and these DSBs are repaired through classical nonhomologous end joining (c‐NHEJ) to generate diverse BCR genomic repertoire.

The AID protein, belonging to the APOBEC family, assumes a crucial role during the B‐cell precursor phase by eliminating polyreactive B cells from the immune cell reservoir.^45^ Its primary function involves the induction of point mutations. Dysregulation of AID can result in an elevated mutation burden, translocation events, genomic instability, and the development of lymphoma^46^ (Figure 2). Consequently, the expression and activity of AID are meticulously regulated through a series of molecular mechanisms. AID, an enzyme specific to B cells, is capable of catalyzing the conversion of single‐stranded DNA and also initiating gene conversion of Ig genes in avian species. The uracil generated by AID is recognized as a form of base damage and subsequently triggers the base excision repair (BER) pathway, or it is recognized as a U:G mismatch and subsequently triggers the mismatch repair (MMR) pathway.^18^, ^47^ In the majority of cellular contexts, these types of damage can be effectively repaired with great accuracy through the BER and MMR pathways (Figure 3). However, in activated B cells, the repair of uracil generated by AID is prone to high levels of mutability. During SHM, point mutations primarily occur in the variable (V) region, while during CSR, DNA DSBs are predominantly generated in the switch (S) region. During the repairment of uracil residues, single‐strand DNA breaks are generated and subsequently converted to DSB. The formation of two DSBs in different S regions results in DNA recombination (Figure 1B). After that, mature B cells have the potential to generate any Ig isotypes, and the direction of conversion is determined by accessibility of the S region, which in turn is mediated by transcription across the Ig locus under the influence of cytokines secreted by local T cells and other related cells.

The occurrence of unrepaired or improperly repaired DSBs in DNA can activate cell cycle checkpoints, leading to cell death, making it the most lethal form of DNA damage in the genome.^48^ The repair of DSBs is a necessary process for generating diverse antigen receptors and preventing the development of lymphoid malignancies, such as B and T cell leukemias and lymphomas. Repair of these DSBs through classical nonhomologous end joining (c‐NHEJ) generates diverse BCR genomes, enabling efficient humoral immunity (Figure 3). In the cells of humans and other mammals, the Ku70/Ku80 and XRCC4/DNA ligase IV (Lig4) complexes typically catalyze c‐NHEJ. In certain circumstances, DNA‐PKcs and Artemis can also catalyze c‐NHEJ.^49^, ^50^ The initiation of NHEJ occurs when Ku binds to the broken DNA ends, and with the assistance of DDR factors, re‐ligates the broken DNA ends, protecting them from nucleolytic degradation. This process results in minimal sequence deletions or insertions and can occur at all stages of the cell cycle, but is prone to errors.^49^ The terminal joining (A‐EJ) can catalyze robust CSR in the absence of c‐NHEJ.^48^ The mechanism of A‐EJ is not well understood, but it is defined as a pathway that occurs independently of classical NHEJ. Recent research has suggested that A‐EJ may potentially rival c‐NHEJ in the process of DSBs.

The regulatory mechanisms of CSR are not yet fully understood. Research suggests that CSR is likely activated by B cells through BCR signaling.^43^ At present, it is not definitively established whether the BCR signal alone is capable of triggering CSR, or if the initial activation of B cells is essential and requires T cell signaling to induce CSR. Additionally, auxiliary signals from the CD40 and toll‐like receptor (TLR) signaling pathways also play a role, and multiple auxiliary signals may interact with the primary signal to enhance the robustness of the CSR response.^51^, ^52^, ^53^ Studies have shown that signaling through the most important receptors, CD40, aided by innate receptor TLR4 or T cell help, can induce CSR within 3 days in response to LPS.^54^, ^55^, ^56^

Remarkably, some intrinsic checkpoints that monitor the capacity of B cells to induce CSR have been identified.^57^ Tumor necrosis factor (TNF)‐receptor‐associated factor (TRAF) 3 and 4 were found to enable CSR in the absence of costimulation. TRAF3 restricts the activation of NF–κB and thus limits BCR signal strength and the ability of B cells to induce CSR. Similarly, some negative regulators of B cell CSR have also been identified. Poly(ADP) ribose polymerase 3 (Parp3) promotes the formation of ADP‐ribose on the receptor protein and controls DNA repair, transcription, and chromatin remodeling, thus inhibiting the excessive accumulation of AID at the S region and negatively regulating CSR.^58^ Aryl hydrocarbon receptor, on the other hand, controls B cell functions by limiting the expression of AID.^59^ Another protein called SANT and BTB domain regulator of CSR (SANBR) has been reported to inhibit CSR.^60^ The BTB domain of SANBR mediates its homodimerization and interaction with corepressor proteins such as HDAC1 and SMRT. Thus, a hypothesis is raised that SANBR, together with other BTB family proteins, regulates CSR transcription and affects antibody diversification. Nucleoside diphosphate kinase (NME) family proteins have been shown to have an important housekeeping function in catalyzing the phosphorylation of nucleoside diphosphates.^61^ NME1 has nuclease activity and the knockdown of this protein results in increased CSR.^62^ NME2, however, is thought to play a key role in binding telomeric DNA of S region and G‐quadruplex (G4) DNA sequence.^63^, ^64^ Contrary to the effect of NME1, knockdown of NME2 leads to an increased level of CSR.

In addition to cytokines, certain transcription factors also play a role in CSR. Transcription factors are a class of proteins that have the ability to bind to double‐stranded DNA, as opposed to single‐stranded DNA, and their activities have the potential to impact the process of transcriptional activation.^65^ CSR is initiated by AID, and the transcription factor E2A forms a complex with AID, as well as the transcription factors PAX5, ETS1, and IRF4, at the critical sequences of the Igh locus for CSR in B lymphocytes.^66^, ^67^ Unlike other transcription factors, E2A can also bind to single‐stranded DNA with sequence specificity, and one of its binding sites, µE2, demonstrates a marked inclination towards a specific strand of the intronic enhancer located within the Igh locus. Furthermore, it has been found that E2A can cleave single‐stranded DNA. Studies have shown that E2A not only plays a role in AID targeting antibody genes but may also contribute to the precise localization of AID on the deamination sites for CSR and SHM.

Additionally, epigenetic modifications, such as histone modifications, DNA methylation, and noncoding RNA (ncRNA), also play important roles in antibody maturation during the CSR process.^68^ Specific chromatin modifications can recruit AID, as well as other components of CSR. Changes in chromatin structure occur through histone modifications. Histone modifications can also aid in recruiting AID to Ig loci, in conjunction with DNA primary and tertiary structure. Potential DNA methylation and alternate chromatin modifications, along with ncRNA, collectively regulate the recruitment of AID and stabilize DNA repair factors.

Finally, due to the significant role played by AID in the CSR process and its lack of target sequence specificity in DNA mutation, strict regulation is evidently required to understand how to modulate its activity and focus it on Ig genes in order to avoid severe side effects.^69^ The activity of AID appears to be controlled at multiple levels, including expression, nuclear import, posttranslational modifications, target recruitment, and degradation. For instance, the activity of AID can be regulated through phosphorylation at different sites, with its CSR and recombination‐promoting activity being inhibited by phosphorylation at serine 3.^70^, ^71^ The activity of AID can also be controlled through its subcellular and subnuclear localization. Currently, a major challenge remains in understanding how various protein factors that interact with AID, various chromatin marks, or various cis‐regulatory sequences that control AID accessibility within the Ig HC or LC loci collectively mediate this regulation.

CSR is a multifaceted process initiated by AID and subject to regulation and control by various factors. These factors can impact the activity of AID, the process of CSR, and the induction of CSR in B cells, among other aspects. Many areas within this field require further investigation, such as the specific regulatory mechanisms governing CSR. These factors may potentially influence the body's immune response, such as its antiviral and antibacterial capabilities.

### 2.3 The role of Ig CSR in antimicrobial and viral resistance

**FIGURE 4:**
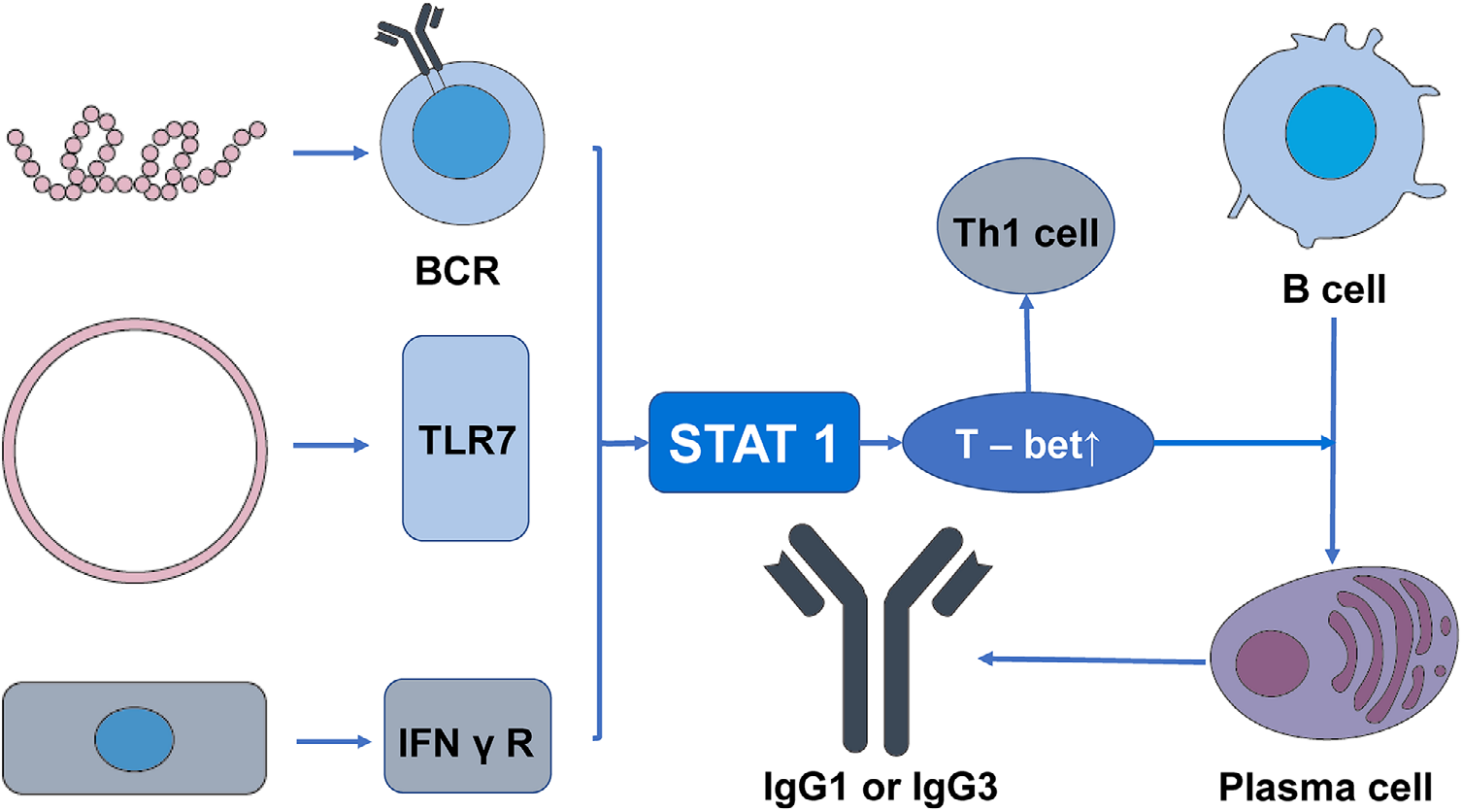
The partial mechanisms of CSR involvement in the antiviral process are illustrated in the diagram. Viral proteins bind to the BCRs of follicular B cells, while viral RNA binds to TLR7. Viral infection can also induce the production of IFN γ through interaction with different cell types binding to B cell IFN γ R.

CSR also plays an important role in the process of combating viral and bacterial infections. Specific cytokines are essential in the process of B cell differentiation as they provide guidance for CSR. For instance, in mice, the IFNγ/signal transducer and activators of the transcription 1 (STAT1) pathway can promote the expression of IgG2a antibodies while inhibiting the production of IgG3, IgG1, IgG2b, and IgE. Conversely, IL‐4 activates transcription factors such as STAT6 to promote the expression of IgG1 and IgE.^72^ In the process of antiviral immune response, the most significant and effective isotype is IgG.^73^ In this procedure, the significance of T‐bet, which is expressed in T cells, has been demonstrated.^74^ The transcription factor T‐bet plays a crucial role in promoting Th1 immune responses and suppressing Th2, and Th17 programs, regulating the differentiation of CD8^+^ T cells, and participating in the mediation of their maturation process. It is also essential for IgG class switching in B cells.^74^, ^75^, ^76^ In B cells, the activation of T‐bet is modulated by various factors, such as BCR, IFNγR, TLR 7, CD40, and the activation of STAT1. In the context of the antiviral immune response, viral proteins and RNA interact with the BCR of follicular B cells and TLR7. Furthermore, viral infection can stimulate the production of IFNγ, which binds to B cell IFNγR. These factors, in the context of viral infection, trigger the activation of STAT1, elevate T‐bet expression, and subsequently promote the production of IgG1 or IgG3 in the human body^77^ (Figure 4). CSR also plays a significant role in the body's defense against bacterial infections. The human gastrointestinal tract harbors trillions of symbiotic microorganisms that contribute to physiological functions such as nutrient absorption and barrier protection, establishing a symbiotic relationship with the host.^78^, ^79^ However, this necessitates that the intestinal immune system must tolerate symbiotic bacteria while preventing pathogen invasion, as dysbiosis can lead to inflammation and various intestinal and systemic diseases.^80^ The process is significantly influenced by humoral immunity, which involves the production and secretion of Igs in the intestinal mucosa.^81^ These Igs play a key role in promoting tolerance to symbiotic bacteria and influencing the microbial composition through noninflammatory mechanisms.^82^ IgA is the primary antibody isotype found on mucosal surfaces, with additional antibody isotypes also playing a role in intestinal humoral immunity.^83^ IgM is effectively transported and excreted into the intestinal lumen, akin to IgA and contributes to the functioning of the immune system.^84^ Studies have shown that in the normal gut, the number of IgG plasma cells is limited, while in individuals with intestinal inflammation, the expression of IgG cells in the mucosa is increased.^85^, ^86^, ^87^, ^88^, ^89^ The class switching of IgA in the intestine requires coordinated interactions among intestinal epithelial cells, dendritic cells, macrophages, and regulatory T cells, inducing B cell class switching in a steady‐state environment rich in IL‐10 and transforming growth factor (TGFβ).^90^ The mechanism by which the immature B cells in the intestinal tract are regulated to undergo class switching to produce IgG plasma cells and memory B cells remains unclear.^91^, ^92^

Hence, certain factors influence the types and quantities of antibodies produced, regulate humoral immunity, and consequently affect the body's defense against pathogens such as bacteria and viruses by guiding and modulating CSR. Further research is required to delve into these mechanisms and gain a deeper understanding of how CSR regulates the secretion of each type of antibody.

## 3 REGULATION OF IG CSR

Various types of Ig have different regulatory mechanisms in the CSR process, and abnormalities in this process often lead to certain diseases. Understanding the mechanisms and roles of these diseases can aid in exploring treatment methods.

### 3.1 Regulation of IgM

IgM is the initial Ig synthesized in response to immunization. The nomenclature of this entity originates from its original characterization as a large molecular weight (900,000 Da) massive spherical protein (M).^93^ There are two different isotypes of IgM, a pentameric form found in the intravascular spaces and a monomeric form located on the surface of B cells.^94^ Plasma cells are produced in two key locations in response to the major group of antigens known as thymus‐dependent (TD) antigens: in the GC of lymph nodes and spleen, and in mucosa‐associated lymphoid tissue (MALT). Most antibodies are typically produced in response to TD antigens. However, certain antigens can activate B cells without the need for assistance from T cells and thus are known as thymus‐independent (TI) antigens. Polysaccharide components of bacterial capsules are one of the clinically significant TI antigens.^95^ Responses to TI antigens are often fast and almost entirely involve the production of IgM antibodies. This IgM may recognize the antigen and activate the complement system, providing critical early defense against many bacterial infections, even in persons who have T cell defects or lack T cells entirely. In contrast, TD‐antigen is recognized and processed by BCR‐mediated endocytosis, presented on MHC II, and recognized by TCR on CD4^+^ T helper (Th) cells.

Interactions between CD4^+^ T and B cells trigger Ig class switching and GC formation in B cells.^96^ CD40L is expressed on the surface of Th cells and interacts with CD40 receptors on B cells. Following this, Th cells secrete cytokines that assist and determine Ig class switching. CD40 signaling initiates the process of Ig CSR by activating the NF‐κB/IκBα complex via the NF‐κB essential modulator (NEMO)–IκB kinase (IKK) complex. With the help of proteases, IκBα is degraded into P50/P65 and finally activates AID, uracil DNA glycosylase (UNG), and B cell‐specific DNA repair enzymes to mediate Ig CSR and SHM.^97^ Defects in antibody production and diversification can be clinically manifested in various immunodeficiency diseases, and uncontrolled antibody diversification can lead to B‐cell lymphoma.

IgM serves as the primary Ig that provides early defense in many immune processes. If obstacles arise during the process of antibody diversification, abnormalities in various types of Ig proteins may occur, leading to the development of multiple diseases.

### 3.2 Regulation of IgD and its implication in diseases

First discovered in 1965, Rowe and Fahey named it IgD because of its distinctive (D) properties compared with other Igs. The structures of IgD Cδ1 and Cδ2 are similar to the C region of other Ig isotypes.^98^, ^99^ However, Cδ3 is a unique domain that lacks seven proline residues to induce the folding of polypeptide skeleton and has two additional N‐linked glycosylation sites. The lengthy hinge region of human IgD features a flexible T‐shaped structure, which permits the two Fab “arms” to spin on each side of the Fc segment, allowing antigen binding.

Immature B cells exclusively express IgM on the cell surface, whereas mature B cells express both IgM and IgD. The rearranged VDJ DNA is transcribed alongside the two nearest C_H_ region genes, Cµ and Cδ. The initial transcript, which contains both Cµ and Cδ RNA, can be spliced in two distinct ways, known as alternative splicing, to produce either a VDJ‐µ or a VDJ‐δ mRNA. As a result, both mRNAs are synthesized by a single mature B cell. The two mRNAs are subsequently translated in the rER to produce either a µ or a δ polypeptide. Individual H‐chain polypeptides join with the L chain to create IgM or IgD, which then traffic to the cell membrane to form mIgM and mIgD. As a result, a mature B cell expresses both IgM and IgD at the surface with identical antigen specificity. IgD can also exist in a secreted form. Secreted IgD is generated by plasma cells rather than mature B cells. IgD might also bind receptors on the surface of basophils, mast cells, and monocytes, which probably enhance their surveillance and participate in antibacterial roles.^98^

It is worth noting that a subgroup of B cells producing IgD but not IgM after undergoing an unusual type of CSR and high level of SHM has been recently discovered.^6^ These IgM^−^IgD^+^ B cells are abundant in respiratory tissues with exocrine glands, but are seldom found outside these regions.^28^ One possible mechanism is that these IgM^−^IgD^+^ B cells are generated through TD‐ and TI‐pathway and subsequently transformed into IgD‐secreting plasma cells. Mucosal DCs provide antigens to active Th cells and aid follicular IgM^+^IgD^+^ B cells in IgM‐to‐IgD CSR via a TD pathway involving CD40, CD40L, IL‐2, and IL‐21. Furthermore, other immune cells are likely to release B cell activating factor (BAFF), proliferation‐inducing ligand (APRIL), IL‐2, and IL‐15, which promote B cell class switching from IgM to IgD via a TI pathway. These IgM^−^IgD^+^ B cells have the potential to develop into plasma cells that secrete IgD directed against respiratory antigens. Recently, Xu et al.^100^ reported that a novel pathway involving zinc finger protein (ZFP) 318 might participate in the induction of IgD. Downregulated ZFP318 phosphorylates Rad52, which then recruits Sµ and σ*δ*, resulting in alternative splicing towards IgD. Rad52 knockout mice abolish IgD CSR in vitro and in vivo while the same scenario is also observed in Rad52 knockout human B cells.

IgD serves as a marker for mature B cells and can be derived from IgM through processes such as T‐cell‐independent conversion. It may play a regulatory role in the antibacterial processes of certain immune cells.

### 3.3 Regulation of IgA

IgA is characterized by its key role in mucosal immunity. Human IgA is divided into two subtypes, IgA1 and IgA2, which differ in the structure of the hinge region and the number of glycosylation sites. IgA1 accounts for 93% of all IgA and is mostly found in the serum, whereas IgA1 and IgA2 are equally distributed in mucosal tissue.^101^ IgA is a monomeric molecule in serum but exists as a dimeric molecule in external secretions. However, the function of serum IgA is less well‐studied compared with secretory IgA. Both TD and TI pathways assist in the production of secretory IgA locally in gut‐associated lymphoid tissue.^102^ During TD responses, IgA CSR is activated through AID‐ and NF‐κB‐dependent pathways, as described above. When TI antigens trigger B cells, extensive BCR cross‐linking or TLR stimulation induces NF‐κB instead.^103^, ^104^ Importantly however, both TD and TI pathways are relevant but not essential for IgA CSR.^105^, ^106^ A third pathway that can activate NF‐κB and trigger recombination of IgA is the binding of an APRIL or BAFF to the transmembrane activator and calcium modulator and cyclophilin ligand interactor (TACI).^107^, ^108^, ^109^ Both TACI‐ and APRIL‐deficient mice have decreased levels of serum IgA but no changes in IgG levels, indicating that they play distinct roles in promoting IgA synthesis.^110^, ^111^, ^112^, ^113^ Moreover, since IgA is mainly produced and performs its function in an intestinal niche, one reasonable assumption is that commensal‐bacteria‐derived butyrate might influence IgA CSR.^114^ Isobe et al.^115^ observed that butyrate‐induced IgA responses are important for maintaining barrier function in the gut and preventing local autoinflammatory responses.

TGF‐β is perhaps the most essential factor in driving IgA production.^116^ TGF‐β activates SMAD2/3 and recruits SMAD 4 to exert its function (Figure 2A). A range of different cytokines regulate gut IgA synthesis, most likely through enhancing plasma cell survival and differentiation. Some interleukins, including IL‐4, IL‐5, IL‐6, IL‐10, IL‐15, and IL‐21, have been shown to play a role in IgA CSR.^117^, ^118^

Furthermore, the regulation of IgA is associated with the RUNX protein, which plays a crucial role in autoimmune diseases. Mammals possess three Runx genes, among which Runx1 is significantly implicated in the process of hematopoiesis and is frequently associated with the development of leukemia in humans.^119^, ^120^, ^121^ Runx2 is essential for skeletal development, and haploinsufficiency of this gene leads to craniofacial abnormalities.^122^, ^123^ Runx3, on the other hand, acts as a tumor suppressor in gastric cancer.^124^ Research has shown that the loss of Runx3 protein is related to DC functional defects, as well as the onset of colitis and symptoms resembling asthma.^125^, ^126^ Runx3 positively regulates the expression of CD103 in various cell types, and mucosal CD103^+^ dendritic cells have the capacity to promote the generation of regulatory T cells via TGF‐β1 and RA‐dependent pathways, which play a crucial role in the CSR of IgA.^127^, ^128^ Therefore, it can be concluded that Runx3 functions as a positive regulator of IgA CSR within DCs. Additionally, several studies have demonstrated that Runx transcription factors synergistically enhance transcription in response to TGF‐β family signaling in collaboration with Smad proteins. Examination of the manifestation of different Runx proteins in I.29, CH12, and splenic B cells when subjected to IgA‐stimulating conditions suggests that Runx3 plays a pivotal role in the process of IgA CSR.

IgA is a crucial component of mucosal immunity, and its production and regulation may be associated with TGF‐β and RUNX proteins.

### 3.4 Regulation of IgE

IgE, also known as reaginic antibody, has a unique role in parasite defense. IgE is produced through classic TI and TD pathways. Berkowska et al.^129^ found that memory B cells can be divided into GC‐dependent (TD) CD27^+^IgE^+^ and GC‐independent (TI) CD27^−^IgE^+^ B cells, and, foreseeably, in patients with CD40 ligand defect, CD27^+^IgE^+^ B cells are difficult to detect while CD27^−^IgE^+^ B cells compensate for nearly 70% IgE‐secreting cells. IL‐4 and IL‐13, the key cytokines participating in type II inflammatory responses in parasite infections, promote expression of ε gene and IgE CSR.^129^ Upon IL‐4 binding to IL‐4Rα (JAK1), the IL‐4/IL‐4Rα complex will recruit and combine with a second receptor chain, either IL‐2Rγc (JAK3) in lymphocytes or IL‐13Rα1 (JAK2) in nonhematopoietic cells. Lymphocytes express very low levels of IL‐13Rα1, while nonhematopoietic cells express the opposite type. After dimerization of IL‐4R and cross‐phosphorylation of JAK, the downstream molecule, activating STAT6, is phosphorylated, dimerizes, and then travels to the nucleus to alter gene transcription.^130^ IL‐13 induces a lower amount of IgE than IL‐4, but those antibodies exist longer, suggesting IL‐13 plays an important role in the production of IgE.

In addition, DOCK8 and STAT3 are involved in the differentiation of IgE‐producing plasma cells. CpG island, together with its binding protein TLR9, recruits MYD88, Pyk2, and Dock8. MYD88 assists in the phosphorylation of Pyk2 and then promotes the phosphorylation of Dock8. Phosphorylated Dock8 recruits Lyn, which in turn phosphorylates Igα/Igβ, the key peptide in BCR signal transduction, activating the Syk–STAT3 pathway, which, finally phosphorylates STAT3 to promote IgE production.^131^ IL‐21, however, activates STAT3 directly after binding with IL‐21R. In humans, IL‐21 and IL‐4 are synthesized together and promote the secretion of IgE after CD40L stimulation.^132^ When STAT3 is inhibited by AG490, the process of transformation of GC B cells into preplasmablast is also prevented. Ding et al.^133^ also found that CD40L could greatly upregulate the duration and amplitude of JAK/STAT3 signaling triggered by IL‐21. Further research demonstrated that anti‐CD40, IL‐4, and IL‐21 stimulation promote more secretion of IgE than anti‐CD40 and IL‐4 alone, suggesting a critical role of IL‐21 in the production of IgE.^134^

IgE is a hallmark component in the immune response against parasites, with DOCK8 and STAT3 involved in the differentiation of IgE‐producing plasma cells, while cytokines such as IL‐13 and IL‐21 play crucial roles in its production. Disruption of these components can impact IgE production, leading to a range of diseases.

### 3.5 Regulation of IgG

IgG is the most abundant Ig in blood and other body fluids. IgG has four subtypes, and, according to their abundance in serum level, namely IgG1, IgG2, IgG3, and IgG4, in descending order.^135^ Each IgG subtype is encoded by a different C region gene and has unique biological and functional properties. Since IgG1 accounts for nearly 66% of total IgG, IgG1 deficiency is often identified as a decrease in total serum IgG level. Serum level of IgG2 reach their normal level of adults at 5−10 years of age. IgG3 is sensitive to proteolytic degradation and therefore has the shortest half‐life.^136^ IgG4, however, has distinct properties and lowest serum level among other IgGs. Analysis of next‐generation sequencing of antibody repertoires identified differences in SHM level across the different Ig types and found unique patterns of class switching between subtypes. Kitaura et al.^137^ observed a high frequency of switching from IgM/IgD to IgA1 or IgA2. The frequency of switching from IgG3 to IgG1 (51.9%) and IgG2 (53.7%) is almost equal, whereas only a small proportion of IgG3 switches to IgG4 (6–7%). In addition, CSR from IgG1 to IgG2, IgA1 to IgA2, IgG2 to IgA2, IgG2 to IgG4, and IgG4 to IgA2 also occur in healthy people. Therefore, IgG3 deficiency usually combines with decreased serum levels of other IgGs.

According to the updated classification of IgG defects, three immunodeficiencies are well defined^138^: isolated IgG subclass deficiency, IgA with IgG subclass deficiency, and specific antibody deficiency with normal Ig concentration and normal number of B cells. IgG3 deficiency is the most common among all patients within IgG subtype isolated selective deficiency, followed by IgG1 deficiency, IgG2, and IgG4.^139^ Since IgG2 subclass antibodies account for most if not all of polysaccharide‐specific responses, IgG2 deficiency is most often associated with recurrent bacterial infections while IgG4 deficiency is commonly combined with deficiency of IgG2.^140^ A combined deficiency of IgG4, IgG2, and IgA has also been reported.^141^ Isolated IgG4 deficiency, on the other hand, is much less common. However, the pathogenesis of these diseases is still unknown.

## 4 DISEASES RELATED TO IG CSR

The aforementioned information indicates that each type of Ig plays a distinct role in the immune process and is an indispensable component. When their regulatory mechanisms are disrupted, such as in cases of gene defects or deficiencies, the production of Igs can be affected, leading to disease (Table 1). Exploring these diseases through the lens of Ig CSR can assist in identifying treatment targets more effectively, holding significant clinical relevance.

**TABLE 1:** Summary table of diseases related to CSR, including disease name, classification, main features, and associated genes.

	Disease		Key feature	Associated gene	References
IgM	Hyper‐IgM syndrome (HIGM)	HIGM1	Normal or higher serum IgM, extremely low IgG, and almost undetectable serum IgA and IgE levels	CD40L gene	^144^
		HIGM3		CD40 gene	^148^
		HIGM2		AICDA gene	^150^
		HIGM5		UNG gene	^155^, ^156^, ^157^
		HIGM6		IKBKG gene	^158^
		HIGM4		Not well studied	
	Waldenström macroglobulinemia (WM)		An indolent B cell lymphoma of older adults	MYD88 gene	^164^, ^165^, ^167^, ^168^
				CXCR4 gene	^164^
IgD	Hyper‐IgD syndrome (HIDS)		Elevated serum IgD levels, increased circulating IgM^−^IgD^+^ B cells	MVK gene	^172^
IgA	Selective IgA deficiency (sIgAD)		A serum IgA level less than 0.07 mg/mL	Multifactorial	
IgE	Hyper‐IgE syndrome (HIES)	AR‐HIES	Elevated serum IgE level	DOCK8 gene	^186^, ^187^, ^188^
		AD‐HIDS		STAT3 gene	^189^
IgG	Immunoglobin G4‐related disease (IgG4‐RD)		Four potential autoantigens	Not well studied	
	Multiple myeloma (MM)		Disturbance of DSB repairment during B cell CSR	IgH locus	^194^
	Burkitt lymphoma		A non‐Hodgkin lymphoma mainly in children and young adults	MYC gene	^201^

### 4.1 Implication of IgM in diseases

There are two primary reasons for IgM‐induced diseases, namely the lack of Ig CSR due to the affected molecules and the malignant proliferation of lymphoplasmacytic cells and monoclonal Ig.

#### 4.1.1 Hyper‐IgM syndrome

Discovered in 1961, HIGM is one of the classic and well‐studied primary immunodeficiency diseases related to Ig CSR deficiency. HIGM is characterized by normal or higher serum IgM, extremely low IgG, and almost undetectable serum IgA and IgE levels,^142^ and can be divided into six categories according to affected molecules.^143^

HIGM1, also called X‐HIGM, accounts for 70% of all HIGM patients and relates to a defect in CD40L gene, which is located on Xq26.3–Xq27.1 and contains 5 exons and 4 introns.^144^ CD40L is a membrane glycoprotein and homologous to TNF‐α, which interacts with CD40, a member of TNF receptor family, and the loss of this interaction is responsible for the Ig deficiency (Figure 1A). At present, 228 mutation sites are identified and published in the Human Gene Mutation Database (http://www.hgmd.cf.ac.uk/ac/index.php), including point mutation, missense mutation, insertion, deletion, and so on, among which mutations in exon 5 are the most common.^145^ Deletion of four nucleotides in exon 3 causes a frameshift and an earlier termination of translation, but the stability of CD40L and the binding ability of detective antibody do not seem to be affected.^146^ Most of these variants are in the extracellular domain, inhibiting protein expression and interaction with CD40. However, mutations in the transmembrane domain are rarely observed. Palterer et al.^147^ recently reported a case with mutations disrupting the CD40L transmembrane domain and thus causing atypical clinical features.

Another common defect is mutations on the gene coding for CD40,^148^ namely an autosomal recessive form of HIGM (AR‐HIGM) or HIGM3. CD40 is expressed mainly on the surface of B cells. The binding of CD40 and CD40L could stimulate the activation of NF‐κB and promote Ig CSR from IgM to IgG, IgA, and IgE. Mutations affect the interaction of B and T cells and thus result in CSR deficiency (Figure 1A). Lanzi et al.^149^ identified a patient with a missense mutation on the CD40 gene and those misfold CD40 proteins were trapped in the endoplasmic reticulum, which indicates HIGM3 might be a protein transport disorder.

Some defects in CSR‐related enzymes and proteins are also critical to Ig CSR. The AICDA gene, located on 12q13, encodes for the AID enzyme, and its recessive mutations are classified as HIGM2. AID is selectively and transiently expressed in GC B cells^150^ and deaminates cytosine in S and IgV region to uracil. Then, UNG hydrolyses the N‐glycosidic bond, releases the free uracil base, and forms single‐stranded DNA breaks, which are subsequently transformed into double‐stranded DNA breaks through MMR.^151^ Breakage of double‐stranded DNA in two S regions is the beginning of DNA recombination and the hallmark of CSR and SHM potential in a single B cell (Figure 1B). Besides, Zahn et al.^152^ reported a case with C‐terminal mutation in AID but have a genetically dominant phenotype. This patient had CSR defects but normal SHM, suggesting the AID C‐terminus might have a unique function in binding CSR costimulatory molecules. Recently, G4 structures have been proven to affect AID targeting.^153^ Qiao et al.^154^ revealed that AID–G4 binding forms oligomers and has a 10‐fold higher affinity for the S region. Moreover, mutations in disrupting this binding significantly impaired CSR without altering the activity of deamination.

As discussed above, mutations on the UNG gene (HIGM5) also are responsible for CSR defects. UNG gene is located on 12q23–24.1 and is considered an autosomal recessive inheritance. UNG is necessary for CSR in B cells, which can splice and repair deoxy‐uridine residues produced by AICDA and promote SHM.^155^ Knockdown of UNG gene or abolishing UNG activity with UNG inhibitor can increase the probability of G to A mutation without affecting the total SHM rate.^156^ However, the role of UNG in CSR and SHM is still very controversial. Rada et al.^157^ showed that, in UNG‐knockout mice, only 1–2% of splenic B cells produced IgG1, which was about 1/14 of the normal mice, while the proportion of IgG3‐expressing B cells was less than 1%, indicating that UNG is critical to CSR. These findings suggest that UNG might have different functions in CSR and SHM.

HIMG6 is associated with recessive mutations on the inhibitor of kappa light polypeptide gene enhancer in B‐cells, kinase gamma (IKBKG) gene.^158^ IKBKG gene encodes NEMO and promotes the release of NF‐κB. NEMO is a component of the IKK, which is a protein kinase complex and contains 4 major parts, IKKα, IKKβ, IKKγ, and IKAP. IKKγ does not have a catalase domain but participates in the formation of IKK and activation of IKKβ and NF‐κB. Stimulating factors activate IKK to induce phosphorylation, ubiquitination, and degradation of IκB, so as to activate nuclear transcription factor and regulate the transcription of corresponding genes (Figure 1A).^159^ NF‐κB then binds enhancer gene sequence (GGGACTTTCC) in Ig κ LC gene and regulates survival, maturation, CSR, and SHM in B cells.

HIGM4, reported by Imai et al.^160^ in 2003, has not been well studied. Most believe that there are selective CSR defects downstream of the activated AID region, while SHM is normal. Other genes related to CSR defects are also identified. Durandy and Kracker^161^ reported patients with HIGM, ataxia‐telangiectasia, and Nijmegen breakage syndrome. The increased radiosensitivity of fibroblasts in such patients is speculated to be related to DNA repair defects. Péron et al.^161^, ^162^ reported HIGM patients with postmeiotic segregation increased 2 mutations, which interferes with DNA repair.

In summary, special cases and detailed studies have promoted further research on the molecular mechanism of HIGM. Endoplasmic reticulum retention of CD40 provides new insights into the pathogenesis of HIGM3, suggesting that intracellular structures related to protein synthesis should be studied. Different roles of AID C‐terminal and UNG in CSR and SHM might reveal a distinctive pathway of CSR. Mutations at different sites of CD40 gene are often associated with other immunological diseases, which need to be further clarified. Genetic technology such as whole‐exome sequencing and CRISPR are critical tools for further scientific research and therapeutic approaches.

#### 4.1.2 Waldenström macroglobulinemia

WM is an indolent B cell lymphoma of older adults, first discovered by a Swedish scientist Waldenström in 1944 and classified as a lymphoplasmacytic lymphoma secreting monoclonal IgM by World Health Organization.^163^ In 2012, Treon et al. showed 91% of WM have mutations on the MYD88 gene (MYD88L265P), while in 2014, Hunter et al. identified another mutation on the CXCR4 gene (CXCR4WHIM) in about 21% MM.^164^, ^165^

TLR is a type I transmembrane protein containing an extracellular domain, transmembrane module, and intracellular Toll‐IL‐1 receptor (TIR) region.^166^ Myeloid differentiation primary response protein 88 (MYD88) gene is located on chromosome 3 and encodes one of the five intracellular adaptor proteins of the TIR. Interaction between MYD88 and TLR activates two independent downstream molecular pathways, interlukin‐1 receptor‐associated kinase 1–4 (IRAK1–IRAK4) and Bruton's tyrosine kinase (BTK) pathways, MYD88 finally activates NF‐κB and contributes to Ig CSR.^167^ The MYD88L265P mutation results in spontaneous homodimerization of MYD88 and recruitment of downstream IRAK1–IRAK4. When the formation of the MYD88/IRAK complex is an uncontrolled, malignant proliferation of lymphoplasmacytic cells and monoclonal Ig appears (Figure 1A).^168^ The CXCR4 gene mainly participates in the homing of CD34^+^ cells and promotes migration, adhesion, and proliferation of tumor cells, thus not being discussed here.

Monoclonal gammopathy of undetermined significance (MGUS) of IgM type (IgM‐MGUS) is considered a precursor for WM.^169^ At least 50% of individuals with IgM‐MGUS already have MYD88 mutations. An additional CXCR4 mutation, which is seen in almost 15−20% of IgM‐MGUS patients, indicates a worse progression.^170^ However, not all IgM‐MGUS patients progressed to WM, there are reports of CLL and non‐Hodgkin lymphoma outcomes.^171^ High serum IgM levels could probably be explained by the same mechanism of MYD88 mutations in WM described above.

### 4.2 Hyper‐IgD syndrome

**FIGURE 5:**
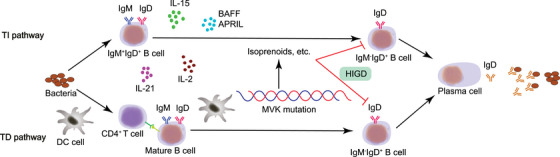
Production of IgM^−^IgD^+^ B cells and IgD antibody. IgM^−^IgD^+^ B cells are produced through TI and TD pathways with the help of DC cells, IL‐2, IL‐15, IL‐21, BAFF, APRIL, and so on, to perform their antibacterial function in mucosal immunity. BAFF, B cell activating factor; APRIL, a proliferation‐inducing ligand; MVK, mevalonate kinase.

HIDS patients have a history of recurrent periodic fever, constitutional symptoms, elevated serum IgD levels, and increased circulating IgM^−^IgD^+^ B cells^172^ caused by mutation in the mevalonate kinase (MVK) gene. The precise pathway through which MVK mutation affects IgM^−^IgD^+^ B cells is still unknown, but one possible process is that IgM^−^IgD^+^ B cells are affected by isoprenoids, one of the mevalonate‐derived products, and normal physiological functions of these B cells are disrupted (Figure 5). Clinical treatments show IL‐1β‐targeting drugs, such as anakinra, are beneficial to HIDS patients, suggesting IL‐1β participates in the pathogenesis of HIDS.^173^, ^174^ Furthermore, the increase in IgD may not be due to the inhibitory effect of CSR on IgD CSR and in vivo secretion, but rather a compensatory response by IgM^+^IgD^+^ B cells due to the absence of other isotype switching.^28^ However, serum IgD levels may be within acceptable limits, and a study has reported that levels of serum IgA, especially IgA1, are increased in HIDS patients.^175^, ^176^

### 4.3 Selective IgA deficiency

sIgAD is a heterogeneous illness with a serum IgA level of less than 0.07 mg/mL.^177^ The mechanisms of sIgAD are most likely to be multifactorial rather than monogenic. Since IgA are generally recognized for their importance in mucosal immunity, microbiota dysregulation is therefore considered as a possible reason for sIgAD. Interestingly, although the level of IgA in the intestinal mucosal is greatly reduced in germ‐free mice, serum IgA levels only decrease to half of the normal quantity in sIgAD.^178^ This is probably because serum and mucosal IgA are produced separately. TGF‐β receptor, another possible pathogenic site in sIgAD, has been reported to be associated with defects in IgA production. Macpherson et al.^179^ showed that SMAD3, 4, and 7 participate in the regulation of IgA‐CSR.^180^ Overexpressed SMAD3 and 4 selectively increase IgA levels and SMAD3‐deficiency mice were identified with no IgA. On the contrary, SMAD7, an inhibitor of TGF‐β, could decrease the IgA level, and, SMAD7‐deficient mice have increased IgA CSR (Figure 1A). Other possible explanations for sIgAD include receptor defects,^102^, ^181^ cytokine network defects,^182^ and microbiota defects,^183^ and so on.

### 4.4 Hyper‐IgE syndrome

HIES, or Job syndrome, was first discovered and described by Davis and Wedgwood in 1966.^184^ In 1972, Buckley et al.^185^ identified elevated serum IgE levels in patients with Job syndrome and named it HIES. Since then, several classic mutations and key proteins have been described.^185^ In 2004, mutations in DOCK8 gene were identified, and, a new concept, autosomal recessive HIES (AR‐HIES), was launched.^186^ By regulating cytoskeletal rearrangement, DOCK8 could affect the migration of dendritic cells and differentiation of lymphocytes. Mutations on DOCK8 gene lead to impaired T cell activation and immune responses, and affect B cell function and Ig production. Dock8 acts as a connector that facilitates the linkage between TLR–MYD88 signaling and B cell activation, a pathway that has been demonstrated to be essential in the production of Igs (Figure 1A).^187^, ^188^ Then, 3 years later, mutations were reported in the STAT3 gene and shown to be related to the pathogenesis of autosomal dominant HIES (AD‐HIDS).^189^ As an important component in JAK–STAT3 signaling pathway, STAT3 participates in regulating cell proliferation, differentiation, and immune regulation. Wesemann et al.^190^ observed a reduced level of antigen‐specific memory B cells and an increased level of immature B cells, suggesting that serum IgE anomalies are associated with impaired B cell maturation. Besides, disrupted IL‐21/IL‐21R signaling pathways also existed in AD‐HIES patients, which affects Ig synthesis and recombination.^191^ Based on the updated International Union of Immunological Societies PID classification, some other mutated genes were classified, such as zinc finger protein 341 (ZNF341), serine peptidase inhibitor kazal type 5 (SPINK5), tyrosine kinase 2 (TYK2), IL‐6 signal transducer (IL6ST), and so on.^138^ TYK2 is a tyrosine kinase and exerts influence on phosphorylation and dimerization of STAT3, and thus regulates Ig isotype switching. However, the function of TYK2 in Ig switching is still controversial. Kreins et al.^192^ reported eight TYK2 deficient patients but only 1 has elevated IgE serum level. The exact pathways of abnormal IgE production in HIES patients with the latter mutations are still unclear and further research is encouraged.

### 4.5 Immunoglobin G4‐related disease

IgG is categorized into four subtypes: IgG1, IgG2, IgG3, and IgG4, with IgG4 exhibiting the lowest concentration in normal serum, representing only 5% of total IgG levels. IgG4‐RD comprises a cluster of autoimmune conditions characterized by fibrosis and inflammation across various organs and systems. The initial documentation of this disease dates back to 2001 when Japanese researcher Hamano and collaborators identified autoimmune pancreatitis. Subsequent investigations have revealed the presence of IgG4‐RD in solid organs like the liver, kidneys, and lungs. In 2010, a publication in Autoimmunity Reviews officially acknowledged the existence of this ailment. Noteworthy features of IgG4‐RD encompass: (1) notably elevated serum IgG4 levels, (2) the occurrence of numerous IgG4^+^ plasma cells in affected regions, and (3) a favorable response to corticosteroid treatment. Hubers et al. identified a new autoantigen, membrane‐anchored protein A11, in IgG4‐RD, proposing that IgG4 might exert its anti‐inflammatory role by obstructing the interaction between IgG1 and membrane‐anchored protein A11.^193^ Membrane‐anchored protein A11 is a calcium‐dependent phospholipid‐binding protein predominantly found in the cell nucleus. Upon cellular injury, exposed membrane‐anchored proteins are recognized as autoantigens, triggering autoimmune disorders. Presently, four potential autoantigens have been recognized: prohibitin, membrane‐anchored protein A11, laminin 511‐E8, and galectin‐3. The primary autoantigens and immune mechanisms underlying IgG4‐RD remain to be elucidated, necessitating further investigation to pinpoint antigens that elicit immune responses in patients with IgG4‐RD.

### 4.6 Hematological disorders related to Ig CSR off‐targeting

#### 4.6.1 Multiple myeloma

In B cell lymphomas and MM, translocation between IgH locus and other oncogenes is thought to be the genetic basis of B cell malignancy.^194^ Disturbance of DSB repairment during B cell CSR is the key feature of MM.^195^, ^196^ There are seven subclasses of MM, IgG‐MM, IgA‐MM, IgD‐MM, IgM‐MM, IgE‐MM, LC‐MM, and nonsecretory‐MM, according to their secreted monoclonal Ig. Five possible mechanisms have been proposed: CSR translocation, homologous recombination, SHM, V(D)J recombination, and peri‐breakpoint sequence motif.^197^ Major mutations are identified, including t(4;14), t(6;14), t(11;14), t(14;16), and t(14,20), which lead to the overexpression of MMSET, FGFR3, CCND3, CCND1, MAF/Wwox, MAFB, respectively, and all of them are associated with IgH gene and thus contributed to their serum Ig isotypes. For example, in t(4;14), breakpoints are usually found within the IGHM switch followed by the IGHG1 switch region. The IgH gene has two identical enhancers, a powerful 3′‐enhancer, and a weaker Eµ enhancer. The t(4;14) translocation places the MMSET gene under the control of the Eµ enhancer (IgH‐MMSET, der(4)) and the FGFR3 gene under the control of the 3′‐enhancer (IgH‐FGFR3, der(14)). Although both IgG and IgA are observed in MM patients with t(4;14), a link between IgA and t(4;14) is confirmed.^198^ Walker et al.^197^ characterized IgH locus breakpoints in MM and raised a hypothesis for translocation‐based events that might appear early in pro‐B cell level.

#### 4.6.2 Burkitt lymphoma

Burkitt lymphoma is a non‐Hodgkin lymphoma mainly in children and young adults.^199^ MYC gene has been proven to be highly associated with Burkitt lymphoma.^200^ IgH locus (80%) is the most common translocation site for MYC gene, followed by the Igκ [t(2;8)] (20%) or λ [t(8;22)] (20%) LC loci.^201^ Breakpoints in the IgH locus are usually found in the class switch regions. With the help of Ig promoter and enhancer, the repositioned MYC gene has a higher expressed level than normal. Overexpressed c‐myc could induce activation of downstream signaling PI3K–Akt–mTOR pathway, and promote proliferation, differentiation, and apoptosis of malignant cells. Class switching of Igs, on the other hand, are disturbed and yet nearly all tumors express IgM.^202^

## 5 IMPLICATIONS OF IG CSR IN DISEASE AND FUTURE DIRECTIONS

The study of CSR holds great potential in clinical settings. By investigating the regulatory processes of CSR, potential therapeutic targets can be identified. For instance, research has shown that DYRK1A affects CSR activity in both intra‐ and extra‐cellular B cells. It controls CSR through MSH6 phosphorylation and regulates the cell cycle of GC B cells through various cell cycle factors. Drugs targeting DYRK1A have been developed for cancer treatment, and their inhibitory effects on DYRK1A also contribute to alleviating CSR in autoimmune diseases and allergies. Additionally, the loss of Lsh in B cells impairs the typical end‐joining pathway, suggesting that Lsh plays an intrinsic role in B cell development and CSR, providing a potential target for immunodeficiency treatment. Based on the regulation of CSR, novel therapeutic approaches can also be identified. Studies have shown that high‐affinity antigen immunization increases the accumulation and CSR rate of GCs. Boosting the immune system can lead to an increase in the rate of aggregation and secretion of antibodies by genetically modified B cells within GCs. Additionally, the antibody sequences of genetically modified B cells in the spleen display indications of clonal selection. Consequently, it is plausible to consider B cells as dynamic pharmaceutical agents capable of adaptation and evolution.

The regulation of CSR holds significant potential in exploring disease therapies, particularly in the context of humoral immune responses, where G‐rich regions known as G4s exist in the S region of mammals. The refinement of specific G4 ligands for cancer treatment strategies enables the design of targeted drugs that act on specific S regions, thereby modulating the levels of a single type of Ig to control the severity of inflammation.^203^ This approach could serve as a future direction for utilizing Ig CSR in disease treatment.

## 6 CONCLUSION

Antibody diversification and Ig CSR are important parts of humoral immunity in mammals, the presence of antibody CSR is crucial for an effective humoral immune response, as it enables B cells to generate isotype‐switched antibodies that are more efficient in combating infectious pathogens or neutralizing toxins compared with IgM antibodies. Defects in the process of CSR are clinically manifested as various immunodeficiency diseases, and the uncontrollable antibody diversification may lead to B‐cell lymphoma. For example, Ig CSR defects (Ig‐CSR‐Ds) are uncommon primary immunodeficiency disorders distinguished by compromised isotype (IgG/IgA/IgE) switching and normal or increased IgM levels. Furthermore, depending on the particular molecular abnormality, Ig‐CSR‐D may be linked to SHM disorders. Malfunctions in the process of CSR may result in the development of primary immunodeficiency diseases (PID), such as HIGM, which is attributed to mutations in BCR or coreceptor signaling components (such as CD19 or CD40). CSR dysregulation can also result in autoimmune diseases, such as the production of high‐affinity autoantibodies from ectopic GCs, which are characteristic of various autoimmune inflammatory diseases including rheumatoid arthritis (RA). Studies suggest that CSR may also play a role in tumorigenesis and progression. The necessary condition for CSR, noncoding CSR (CSRnc), exhibits distinct patterns of variation in certain subtypes of cancer. The transcription of CSRnc in tumors appears to be caused by infiltration of B cells, and certain cytokines involved in the tumor microenvironment may alter the CSR patterns of infiltrating B cells and ELS B cells. Atypical expression of AID and CSRnc in non‐lymphoma tumor cells could result in cytidine deaminase‐mediated kataegis, a mutation process frequently observed in breast cancer. AID activity contributes to nonspecific SHM and genetic instability in B cell malignancies. In the end, the immune response against tumors is mainly influenced by the equilibrium between tumor‐specific neoantigens and T cells as well as Treg cells. Additionally, the antitumor effect mediated by antibodies can be accomplished through processes such as antibody‐dependent cell cytotoxicity or other mechanisms. However, these studies have certain limitations, as although CSRnc transcription is a necessary condition for CSR, it does not necessarily prove that CSR is actively occurring.

From a clinical perspective, an accurate description of the various Ig‐CSR defects and related diseases is essential since the patients’ outcomes and follow‐up vary from one disease to another. Some Ig‐CSR deficiencies are linked to impaired cellular immune responses, such as Dock8, STAT3, CD40, CD40L, and so on. For these patients, hematopoietic stem cell transplantation (HSCT) is currently the only curative method. Some hematologic malignancies such as MM and Burkitt lymphoma are also associated with Ig‐CSR defects. Translocation of oncogenes to IgH locus and DNA repair factor deficiency results in the occurrence of lymphoma and leukemia. Thus, further studies of Ig CSR and antibody diversification appear to be crucial for potential therapeutic targets and for improving our understanding of the delicate mechanism of Ig diversification.

This review chooses typical diseases in different antibody categories and malignant hematologic disorders to illuminate the latest hypothesis of these diseases. Although much is known about CSR, there are still questions that are left unanswered. Identification of IgG subtypes‐related CSR defects remains difficult. The relationship between IgE CSR defects and allergy are not well known. Genetic basis and molecular mechanism of CSR defects in each disease are difficult and unclear. Thus, studies of Ig CSR defects appear to be critical.

Currently, there are several challenges in CSR research that require further investigation to elucidate certain principles. For instance, the role of G4‐DNA, which can inhibit CSR when bound to various natural or drug ligands, is not fully understood in the S region and S transcripts. The targeting mechanism of AID, which plays a crucial role in CSR, remains unclear despite numerous investigations, as no sufficient or necessary conditions for recruiting AID have been identified. Additionally, the question of why certain long‐range regulatory elements target specific promoters is worth exploring. Transcriptional regulatory elements can exert long‐range control over CSR, but the exact relationship between the signals triggering chromatin looping and its dissolution, as well as the details of the interaction between large chromatin loops and transcription and epigenetic control, are still unknown. Despite these challenges, CSR holds promising prospects for the future. Pharmacological control of CSR could potentially reduce the production of Ig classes that are most favorable for inflammation and the production of IgM. Furthermore, controlling the humoral response, which allows for precise regulation of the secretion of a single category of Ig, could be particularly attractive for promoting or inhibiting inflammation.

In summary, significant progress has been made in the field of CSR mechanisms, regulation, and related disease research. Significant progress has been made in comprehending the transcriptional and epigenetic processes that govern long‐term control of CSR. The clarification of the functionality of recently identified regulatory elements and the involvement of trans‐acting factors has contributed to a heightened level of intricacy in the comprehension of these mechanisms. Research in the field of CSR is of great significance in elucidating cellular‐level physiological mechanisms and identifying therapeutic targets. By regulating CSR, novel therapies can be developed to more precisely treat inflammatory and neoplastic diseases. Furthermore, understanding the regulation and mechanisms of CSR also plays a crucial role in the prevention of autoimmune diseases.

## AUTHOR CONTRIBUTIONS

Jia‐Chen Liu and Ke Zhang wrote the review and drew the figures. Xu Zhang, Fei Guan, Hu Zeng, Masato Kubo, Pamela Lee, Fabio Candotti, Louisa Katherine James, Niels Olsen Saraiva Camara, Kamel Benlagha, Jia‐Hui Lei, Huamei Forsman, and Lu Yang reviewed the paper. Wei Xiao, Zheng Liu, and Chao‐Hong Liu organized and revised the paper. All authors contributed to the review and approved the submitted version.

## CONFLICT OF INTEREST STATEMENT

The authors declare that the research was conducted in the absence of any commercial or financial relationships that could be construed as a potential conflict of interest.

## ETHICS STATEMENT

Not applicable.

## Data Availability

Not applicable.
